# Mechanical Properties of Polypropylene–Cellulose Biocomposites: Molecular Dynamics Simulations Combined with Constant Strain Method

**DOI:** 10.3390/molecules28031115

**Published:** 2023-01-22

**Authors:** Nea B. Möttönen, Antti J. Karttunen

**Affiliations:** Department of Chemistry and Materials Science, Aalto University, P.O. Box 16100, FI-00076 Aalto, Finland

**Keywords:** biocomposites, molecular dynamics, mechanical properties, cellulose, polypropylene, maleic anhydride

## Abstract

The use of biocomposites is increasing due to their recyclability, biodegradability, and decreased CO_2_ emission levels compared to pure polyolefin plastics. Furthermore, suitably engineered biocomposites can provide, for example, superior mechanical properties for various applications. However, the correlations between the atomic-level structure and mechanical properties of most biocomposites are not yet understood. Atomistic molecular dynamics (MD) simulations provide a powerful way to examine the atomic-level structure and mechanical properties of biocomposites. In this study, polypropylene–cellulose biocomposites were examined using maleic anhydride grafted polypropylene (PP-MAH) as a coupling agent. The biocomposites were studied with the Materials Studio program package and COMPASSII force field, using the constant strain approach for mechanical properties. The results were comparable to the experimental literature values, showing that that MD can be applied to study the atomic-level structure–property correlations of polypropylene–cellulose biocomposites.

## 1. Introduction

Composites consist of two or more materials, which differ either physically or chemically from each other. Biocomposites can be made by combining biobased fibers to a matrix. Natural fibers such as cellulose, hemicellulose, chitin, and lignin have shown huge potential in the development of biocomposite materials [1,2]. Fibers are usually collected from trees or crops and the material is added to the matrix material, which is often some kind of plastic, although all-cellulose composites are also being studied [3]. The fibers in the biocomposites can be arranged in different orientations, which affect the mechanical properties of biocomposites [4].

Biocomposites have gained more and more popularity in the past few decades due to growing environmental awareness. Materials that have good recyclability, CO_2_ neutrality, and biodegradability can compete against traditional materials used in different industries [5]. In addition to being eco-friendly, many biocomposites are low-cost and lightweight materials with excellent specific mechanical properties, which makes them good alternatives to glass and carbon fiber composites [5,6]. 

Plastic matrix materials used in biocomposites can be categorized into thermosets and thermoplastics. Thermoplastics have better recycling possibilities compared to thermosets and that is why the use of thermoplastics will likely exceed the use of thermosets in the next years. Polypropylene (PP) is the thermoplastic that shows the best compatibility for matrix material used with natural fibers [7]. PP has excellent mechanical and physical properties [8] together with a relatively low cost [9]. Polypropylene also makes an excellent matrix material for composites due to its low density and relatively good impact resistance [8]. 

Natural fibers used in biocomposites consist mainly of cellulose and lignin [7]. The cellulose-containing fibers are hydrophilic and thus do not bond well with the hydrophobic PP matrix. This is one downside of natural fiber-based biocomposites, but the problem can be solved either by physically modifying the fibers or by using a coupling agent which enhances the fiber–matrix adhesion. Good adhesion enables the matrix material to transfer the mechanical load to the fibers, which strengthens the composite [10]. The coupling agent enhances the composite, giving it a better mechanical strength. One coupling agent group showing good results in cellulose and PP composites is anhydrides [11]. Maleic anhydride can either be added to the composite mixture alone or it can be attached to polypropylene chain, forming maleated polypropylene (PP-MAH). For example, Nachtigall et al. examined the effect of a coupling agent in a polypropylene/wood powder composite, and they found a notable effect [12].

Compared to glass fiber composites, natural fiber composites provide excellent specific strength. While the absolute strength of natural fibers is inferior to glass fibers, their density is low enough to raise the specific strength to the level of glass fiber composites. In addition, natural fiber composites have great flexural modulus and flexural strength [7].

Although biocomposites are becoming more and more popular, the understanding of the materials at the atomic level is still rather limited. When using PP-MAH as a coupling agent, the interactions between the cellulose fiber and the MAH group in PP-MAH are not understood in detail. Atomistic molecular dynamics (MD) simulations offer a possibility to investigate the structures and properties of materials at the atomic level and they have been used to study biocomposites, for example by Ju et al., He et al., and Modi et al. [13,14,15].

Here, we use MD simulations to examine the structures and mechanical properties of polypropylene–cellulose biocomposites with a maleated polypropylene coupling agent. We first investigate the individual components, cellulose fiber, polypropylene, and MAH, separately, after which we study several possibilities of attaching the PP-MAH to cellulose. Finally, MD simulations are carried out to study the mechanical properties of the composite.

## 2. Methods and Models

### 2.1. General Computational Details

The molecular dynamics simulations were performed using BIOVIA Materials Studio, version 20.1. After initial benchmarks on crystalline cellulose and polypropylene with COMPASS [16] and COMPASSII [17] forcefields (Condensed-phase Optimized Molecular Potentials for Atomistic Simulation Studies), the more recent COMPASSII forcefield was chosen for production runs. Electrostatic interactions were calculated using particle–particle particle–mesh (PPPM) as the summation method [18] and all forcefield calculations were carried out with the Fine quality setting (convergence criteria: Energy 10^−4^ kcal/mol; Max. force 0.005 kcal/mol/Å; Max. stress 0.005 GPa; Max. displacement 0.001 Å). Nosé thermostat [19,20,21] and Berendsen barostat [22] were applied in the MD simulations carried out in an NPT environment, using *T* = 298.15 K and *p* = 0.1 MPa (maximum simulation length was 100 ps). All molecular dynamics simulations used a timestep of 1 fs. All geometry optimizations during the building of the structure models were carried out with the steepest descent algorithm. Mechanical properties were calculated using the Constant strain approach implemented in Materials Studio (four steps per each strain, maximum strain amplitude 0.003).

### 2.2. Structure Models for Cellulose

**Figure 1:**
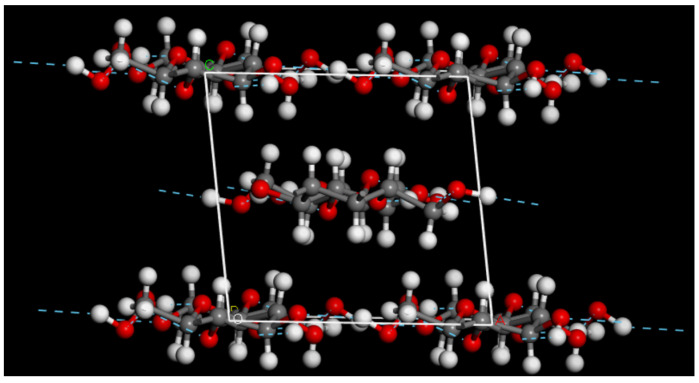
Unit cell of crystalline cellulose Iβ. The unit cell vectors are drawn in white.

The construction of the cellulose structure model started from the unit cell of crystalline cellulose Iβ shown in Figure 1 [23]. Compared to the experimental lattice parameters of Nishiyama et al. [23] (*a*: 8.201 Å, *b*: 10.380 Å, *c*: 7.784 Å, *β*: 96.5°), the optimized parameters using the COMPASSII forcefield are in good agreement *a*: 8.21 Å, *b*: 10.39 Å, *c*: 7.79 Å, *β*: 96.55°. We further tested the force field by cleaving two to four cellulose planes from the crystal structure and checked that they would stay together during geometry optimizations. When the cleaved cellulose planes were solvated in water and the geometry was optimized, the cellulose planes also stayed together. The solvated model of cellulose was created with the help of the Packing task in Materials Studio, aiming for a density of 0.997 g/cm^3^ for the solvent.

**Figure 2:**
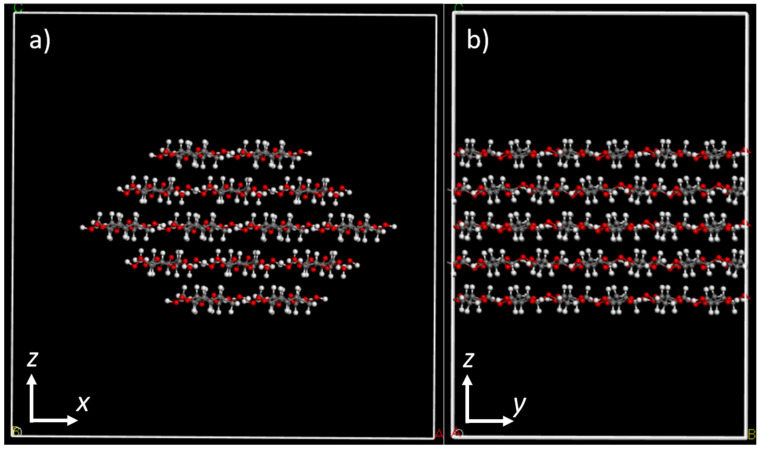
(**a**) Front view and (**b**) side view of a periodic cellulose fibril with 14 cellulose chains and a diameter of 3.33 nm. The dimensions of the simulation box are *a*: 4.5 nm, *b*: 3.1 nm, and *c*: 4.5 nm. The model shown in the figure has 14 × 6 = 84 glucose residues in it.

Next, we cut cellulose fibrils from the bulk cellulose by cleaving the crystal in such way that the fibrils had either 7, 10, or 14 cellulose chains. The fibril model remained periodic in one direction, and a supercell three times the original unit cell size was created in this direction. The resulting one-dimensional fibril models are illustrated Figure 2.

### 2.3. Structure Models for Polypropylene

**Figure 3:**
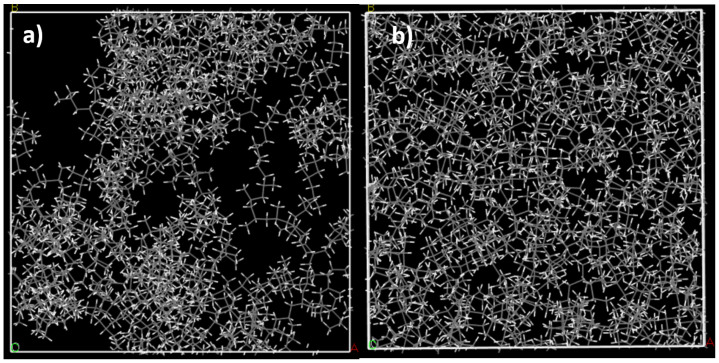
(**a**) Initial model of the amorphous syndiotactic PP containing 10 chains of 50 monomers (box dimension 4.12 nm, density approx. 0.5 g/cm^3^). (**b**) Final model after geometry optimization and annealing cycles (box dimension: 3.46 nm, density approximately 0.84 g/cm^3^).

The model for syndiotactic polypropylene was built with the polymer building tools in Materials Studio. Various polypropylene models were constructed with the Amorphous cell tool, using chain lengths of 18 to 100 monomers and 1 to 50 chains per unit cell. In each case, the unit cell was first optimized with at least 3000 steps of geometry optimization. Next, an MD simulation of 100 ps was performed at 298 K, followed by annealing from 298 K to 600 K and back for 5 times to bring the system to a more uniform configuration. Finally, another 100 ps MD simulation at 298 K was performed and a final geometry optimization with Fine quality setting was used to achieve the model in Figure 3b. The evolution of the structural model during the process is illustrated in more detail in Figures S1–S8 of the Supplementary Material. After the MD simulations, the density of the PP was about 0.84–0.85 g/cm^3^. The literature values of crystalline PP vary, usually between 0.89–0.92 g/cm^3^ [24], while For amorphous PP, a smaller density of 0.85 g/cm^3^ is often quoted, in line with our calculated densities [25].

### 2.4. Structure Models for PP–Cellulose Composite

A molecular model for maleic anhydride (C_2_H_2_(CO)_2_O) was first created, after which the MAH group was connected to a 50 monomer long syndiotactic PP chain with a covalent bond (see Figure S9). The position of the MAH group varied from the middle to the end of the chain and different bonding modes with the PP chain were tested. The final mechanical properties reported here correspond to C–C bonding between PP and MAH. An example of the MAH group positioned in the middle of a 50 monomer long PP chain is shown in Figure S9 in the Supplementary material.

Construction of the PP-MAH–cellulose composite was started by building a 3D box around the cellulose fibril with the Vacuum Slab tool. The size of the vacuum box was determined by calculating the targeted mass percentages of cellulose in the composite. The fibril was moved into the middle of the vacuum box.

**Figure 4:**
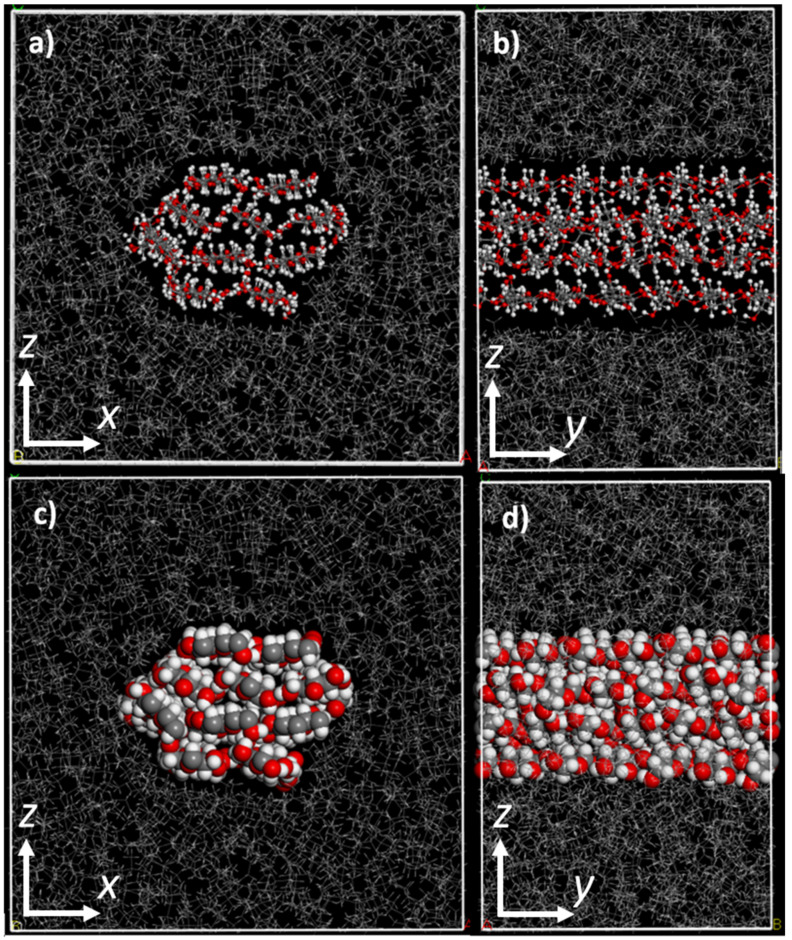
The final structure model of 20 m-% cellulose–PP composite presented in ball-stick (top) and space-filling (bottom) models. (**a**,**c**) show the front view and (**b**,**d**) show the side view of the periodical cellulose fibril. The dimensions of the simulation box are *a*: 5.01 nm, *b*: 3.25 nm, *c*: 5.01 nm. The fibril with diameter of 2.42 nm has 10 chains of cellulose and is surrounded by syndiotactic PP chains of 50 monomers.

First, we built composites without the PP-MAH coupling agent as a point of comparison for the eventual PP-MAH-containing composite. An isosurface was constructed around the cellulose fibril to ensure that when the box is filled with polypropylene, the chains will surround the fibril rather than go through it. The isosurface was created by using the Connolly Surface tool. The vacuum box was then filled with PP chains of 50 monomers by using the Amorphous cell and Packing tools, packing around the isosurface-enclosed fibril. The resulting initial model structure was optimized by at least 3000 steps, followed by 100 ps MD simulation at 298 K and annealing from 298 K to 600 K and back 5 times in 100 ps MD simulations. Finally, another 100 ps MD simulation at 298 K was carried out, followed by a 3000 step geometry optimization. The final structure model is presented in Figure 4.

**Figure 5:**
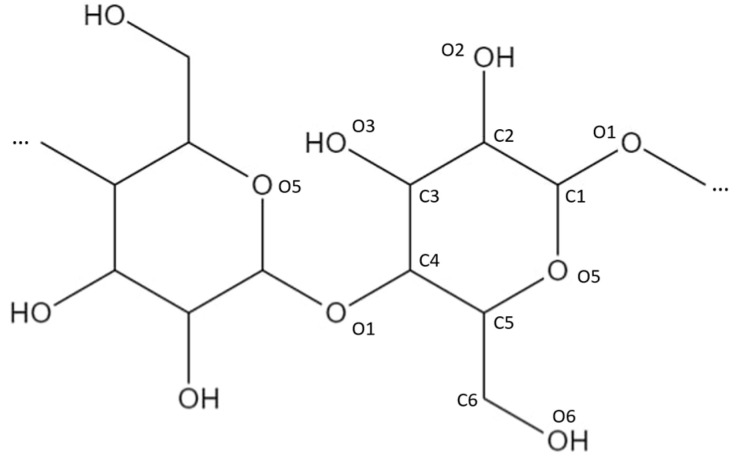
Position and numbering of the carbon and oxygen atoms in the cellulose structure.

**Figure 6:**
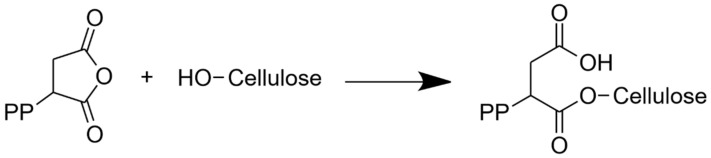
PP-MAH coupling agent bonding to the surface of a cellulose fibril.

For the composite containing PP-MAH, another model from the cellulose 3D box was constructed. The PP-MAH chain was added near the fibril and covalently connected to the side of the fibril by connecting the MAH with one of the OH-groups, O6, in the cellulose chain (Figure 5). This results in an ester group, as shown in Figure 6. A detailed view on the connection of PP-MAH and the cellulose fibril is shown in Figure S10 of Supplementary information. The most likely oxygen atom for bonding with MAH seems to be O6 due to sterical factors (the side of the cellulose fibril is the most available site for connecting with the PP-MAH). Aside from considering sterical arguments, we did not extensively study the other possibilities to form the covalent bond between cellulose fiber and PP-MAH. For such study, it would be most fruitful to utilize QM/MM models where the reactivity of PP-MAH towards the cellulose fiber could be directly investigated.

**Figure 7:**
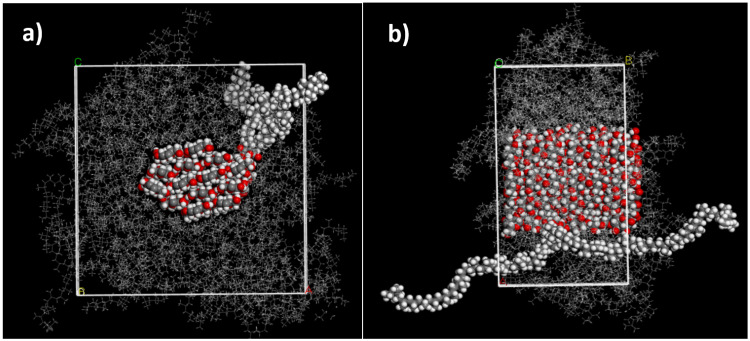
(**a**) Front view and (**b**) side view of 20 m-% cellulose PP-MAH composite (simulation box *a*: 6.04 nm, *b*: 3.24 nm, *c*: 6.04 nm). The fibril with a diameter of 3.00 nm has 14 chains of cellulose. The model has one chain of PP-MAH, where a 50 monomer long syndiotactic PP chain contains one MAH group in the middle of the chain in the same carbon atom where the methyl group is connected. The fibril is surrounded by syndiotactic PP chains that are 50 monomers long. The unit cell of the periodic structure is drawn in white.

The cellulose–PP-MAH structure model was then geometry optimized, after which an isosurface was added around the cellulose–PP-MAH combination and the box was filled with PP chains of 50 monomers. The annealing and structure optimization of the model followed the steps outlined above for the composite without MAH. The final structure model is shown in Figure 7.

## 3. Results

We first calculated the mechanical properties for the pure polypropylene models described above. Table 1 shows the mechanical properties obtained for the amorphous PP unit cells with different numbers of PP chains with varying length. Young’s modulus, bulk modulus, and shear modulus were obtained with constant strain calculations.

Amorphous PP is isotropic and the Young’s modulus in x, y, and z directions should be similar. Table 1 shows that when the PP chain has more monomers and there is a larger number of chains in the cell, the Young’s modulus becomes more isotropic. For example, for 50 monomers and 50 chains, the Young’s moduli vary between 3.1 and 3.4 GPa, while for 100 monomers and 10 chains, they vary between 3.2 and 3.4 GPa. The bulk moduli converge to 2.5–2.6 GPa and shear moduli to 1.3 GPa. The literature values for the mechanical properties of polypropylene (with density 0.89—0.92 g/cm^3^) are: Young’s modulus 1.3 GPa and shear modulus 0.4 GPa [24]. In comparison to the literature values, the calculated Young’s moduli and shear moduli are larger, but not excessively. The structural models used here are, in principle, amorphous, but this is only an approximation, as the calculations do make use of periodic boundary conditions. Size effects probably also play a role, as for a larger box of 10 × 10 × 10 nm filled with PP chains of 100 monomers, a slightly smaller Young’s modulus of 2.4 GPa has been obtained [15].

For pure cellulose Iβ, the calculated Young’s modulus is 37.0 GPa, 136.8 GPa, and 12.7 GPa in the *x*, *y*, and *z* directions, respectively. As expected, the largest Young’s modulus was obtained along the cellulose chains. The experimental Young’s moduli reported for cellulose vary a lot, but for the fully crystalline case, values larger than 100 GPa and even up to 138 GPa have been reported in the direction of the cellulose chains [26,27]. Our calculated value would be in good agreement with the value of 138 GPa. The second largest direction was along the hydrogen bonds within the cellulose planes and the smallest modulus obtained was calculated in the stacking direction of cellulose planes with only weak van der Waals interactions. Experimental Young’s moduli for these two directions vary from 8 to 57 GPa, which is also in reasonable agreement with our results [26]. The calculated bulk modulus for pure cellulose was 17.1 GPa and shear modulus 8.9 GPa. The mechanical properties of cellulose depend strongly on the moisture of the material: a bulk modulus of approximately 10 GPa has been reported for cellulose that is not completely dry [28].

**Figure 8:**
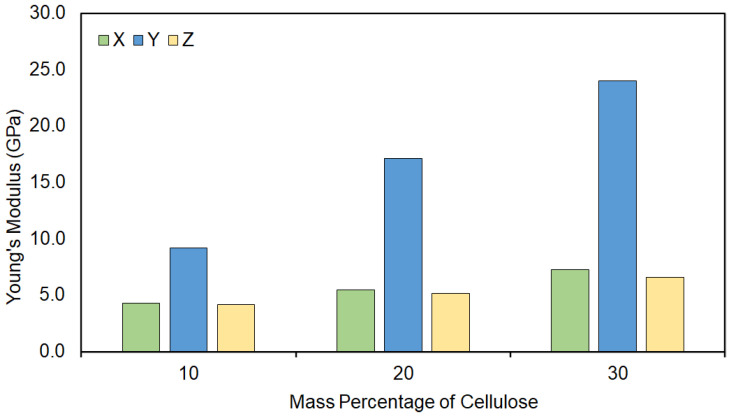
Young’s modulus of a 10 chain cellulose fiber surrounded by 50 monomer long PP chains. Three different models are reported, each with a different mass percentage of cellulose compared to the total mass of the unit cell.

Table 2 shows the calculated mechanical properties for the cellulose–PP composite without the PP-MAH coupling agent. The density of the composite was approximately 1 g/cm^3^. Three cellulose fibrils of different size were used (7, 10, or 14 chains) and different mass percentages of cellulose were used depending on the fibril size. Young’s modulus, bulk modulus, and shear modulus were calculated with the constant strain method. Figure 8 presents the Young’s modulus data for the 10 chain cellulose fiber composite with 50 monomer long PP chains. The cellulose chains run along the *y* direction, and therefore the Young’s moduli in this direction is dominated by the cellulose. The Young’s moduli in the *x* and *z* directions give a more realistic view of the effect of cellulose in the mechanical properties of the polypropylene matrix. As can be expected, the higher the mass percentage of cellulose, the higher the Young’s moduli become. For example, in the model with 14 cellulose chains and 30 m-% of cellulose, the average Young’s modulus in the *x* and *z* directions is 7.5 GPa. This is more than double the value obtained for amorphous PP with a 50 monomer chain (Table 1). The cellulose thus affects the mechanical properties also in directions which are perpendicular to the direction of the cellulose chains.

The mechanical properties of the cellulose–PP-MAH composite are shown in Table 3. Again, cellulose fibers of 7, 10, and 14 chains were used together with PP-MAH with 50 PP monomers. Young’s modulus, bulk modulus, and shear modulus were calculated. When comparing the results of composites with and without PP-MAH in Table 2 and Table 3, we can see that the composite containing PP-MAH seems to have a slightly higher Young’s moduli compared to the cellulose–PP composite: for example, in the model with 14 cellulose chains and 20 m-% of cellulose, the Young’s moduli are approximately 10% larger compared to the composites without the MAH coupling agent. However, the difference is not that large, and a more extensive study on the mechanical properties as a function of MAH m-% and bonding modes with cellulose is needed. Here we aimed for a typical m-% of MAH used in experiments, but possibly, by increasing the MAH m-%, the mechanical properties could be further improved. The magnitude of the MAH coupling agent on the mechanical properties is similar to the previous study of Modi et al. [15], even though a different methodology for obtaining the mechanical properties was used in that study. The absolute values of Young’s moduli are higher here, likely due to the smaller-size models used in this study. Overall, the trends in the mechanical properties obtained with constant strain simulations here are in line with the properties obtained with uniaxial compression simulations, larger models sizes, and a different force field in [15].

## 4. Conclusions

We used atomistic molecular dynamics simulations to study the atomic-level structure and mechanical properties of polypropylene–cellulose biocomposites. Biocomposites with and without maleic anhydride-grafted polypropylene (PP-MAH) as a coupling agent were studied. The COMPASSII force field described the polypropylene and cellulose components of the biocomposite well in comparison to experiments. The constant strain approach provided mechanical properties that were in line with previous experimental and computational studies. Introducing cellulose into the PP matrix resulted in a clear increase in the Young’s moduli: in PP–cellulose composites with 20 m-% of cellulose, the Young’s modulus in the directions perpendicular to the cellulose fibril increases by 60%. The further effect of adding the MAH coupling agent is not as significant: in the model with 14 cellulose chains and 20 m-% of cellulose, the Young’s moduli are approximately 10% larger compared to the composites without the MAH coupling agent. Overall, the used methodology can be applied to study the atomic-level structure–property correlations of polypropylene–cellulose biocomposites. In future studies, the bonding modes between the MAH coupling agent should be studied with quantum chemical methods and further optimization of the coupling agent mass percentage could lead to improved mechanical properties in the biocomposites. Another future direction would be to extend the atomistic models in such way that there would be several fibrils of finite length in the unit cell, enabling comparisons with empirical, macroscopic Halpin–Tsai models of composite materials [29,30].

## Figures and Tables

**Table 1:** Mechanical properties for amorphous polypropylene unit cells with 18 to 100 monomers and 1 to 50 PP chains.

		Young’s Modulus (GPa)				
Monomers	Number of Chains	x	y	z	Bulk Modulus (GPa)	Shear Modulus (GPa)
18	1	1.4	2.4	4.1	3.2	1.3
	5	2.9	2.5	2.5	1.9	1.2
	10	2.5	3.2	3.1	2.1	1.2
30	1	4.5	3.2	4.1	3.0	1.1
	5	3.5	2.7	3.6	2.2	1.2
	10	2.5	2.6	3.5	2.1	1.1
50	1	2.4	2.1	3.3	1.6	1.1
	5	3.2	3.3	3.2	2.4	1.3
	10	3.9	4.0	3.0	2.6	1.3
	50	3.2	3.1	3.4	2.5	1.3
100	1	3.4	3.4	2.4	2.4	1.2
	5	3.3	3.4	3.5	2.4	1.3
	10	3.4	3.2	3.4	2.6	1.3

**Table 2:** Simulated mechanical properties of cellulose–PP composite using different amounts of cellulose chains in fibril and different mass percentages of cellulose. PP chains had 50 monomers in them.

		Young’s Modulus (GPa)				
Cellulose	Mass Percentage	x	y	z	Bulk Modulus (GPa)	Shear Modulus (GPa)
7	10	4.4	10.1	3.8	3.4	1.8
	20	5.1	16.6	5.3	4.9	2.4
	30	4.8	23.6	4.8	4.9	2.5
10	10	4.3	9.2	4.1	3.4	1.8
	18	4.7	15.3	4.6	4.2	2.2
	20	5.4	17.1	5.1	4.9	2.5
	28	7.3	24.0	6.6	5.8	3.2
14	20	5.5	16.4	5.1	4.9	2.5
	30	7.7	25.9	7.2	6.4	3.3

**Table 3:** Simulated mechanical properties of the cellulose–PP-MAH composite using different amounts of cellulose chains in fibril.

				Young’s Modulus (GPa)				
Cellulose	m-% (Cellulose)	m-% (PP-MAH)	m-% (MAH)	x	y	z	Bulk Modulus (GPa)	Shear Modulus (GPa)
7	20	3.4	0.15	5.9	18.0	5.5	5.2	2.6
10	18	4.1	0.18	5.3	15.3	5.3	4.5	2.4
14	20	3.2	0.14	5.9	17.0	5.5	5.2	2.7

## Data Availability

Not applicable.

## Supplementary Materials

The following supporting information can be downloaded at: https://www.mdpi.com/article/10.3390/molecules28031115/s1, Figure S1: Initial structural model of polypropylene generated with Materials Studio; Figure S2: Structural model of polypropylene after geometry optimization; Figure S3: Polypropylene structural model after NPT molecular dynamics run at 298 K; Figure S4: Evolution of the density of the polypropylene structural model during the NPT molecular dynamics run at 298 K; Figure S5: Polypropylene structural model after several annealing cycles 298 K to 600 K and back; Figure S6: Evolution of the density of the PP structural model during annealing cycles; Figure S7: Polypropylene structural model after final NPT molecular dynamics run at 298 K; Figure S8. Evolution of the density of the polypropylene structural model during the final NPT molecular dynamics run at 298 K; Figure S9: The position of MAH in 50 monomers long PP chain is in the middle of the chain and in the same carbon atom as the methyl group; Figure S10: Side view of PP-MAH, with one MAH group in the middle of the 50 monomers long syndiotactic PP chain and in the same carbon atom as the methyl group.
