# Supplementary material for: Mechanical Properties of Polypropylene–Cellulose Biocomposites: Molecular Dynamics Simulations Combined with Constant Strain Method
Source: Molecules. 2023 Jan 22;28(3):1115. doi: 10.3390/molecules28031115 (PMC9921334; doi:10.3390/molecules28031115)
Supplement: Supplementary file 1 [file molecules-28-01115-s001.zip › molecules-2154430-supplementary.pdf]

Supplementary Materials for

Mechanical Properties of Polypropylene-Cellulose Biocomposites: Molecular Dynamics Simulations Combined with Constant Strain Method

Nea B. Möttönen and Antti J. Karttunen*

Department of Chemistry and Materials Science, Aalto University, 00076 Aalto, Finland. Email: antti.karttunen@aalto.fi.

Step-by-step example of generating the polypropylene structural model with the COMPASSII forcefield.

Technical details of the process are given in the main text and supporting figures are given here.

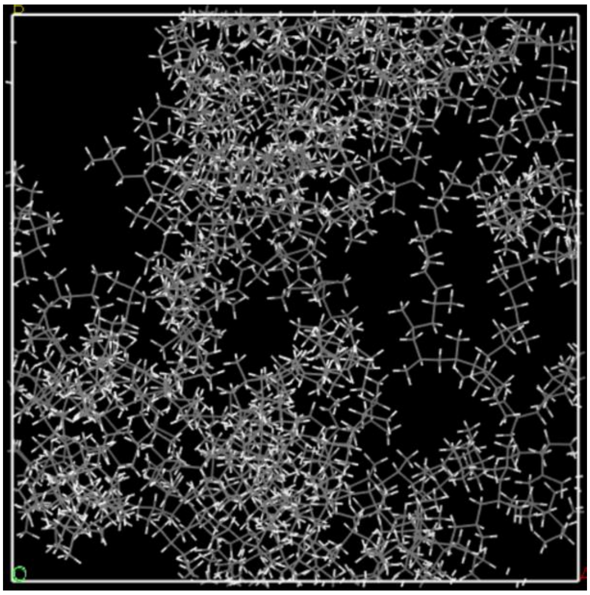

Figure S1. Initial structural model of polypropylene generated with Materials Studio (density 0.500 g/cm^3).

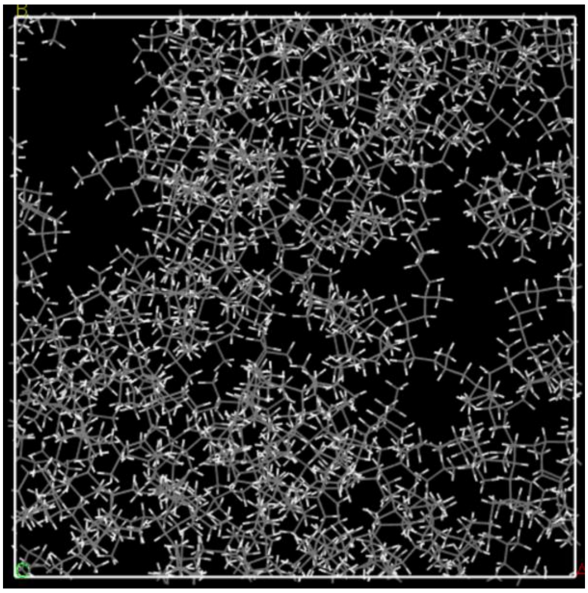

Figure S2. Structural model of polypropylene after geometry optimization (density 0.500 g/cm^3).

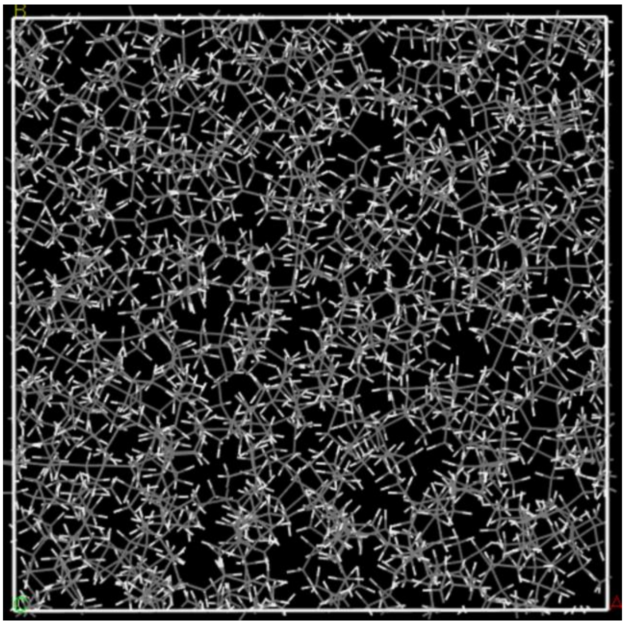

Figure S3. Polypropylene structural model after NPT molecular dynamics run at 298 K, ending up in a density of approximately 0.84 g/cm^3.

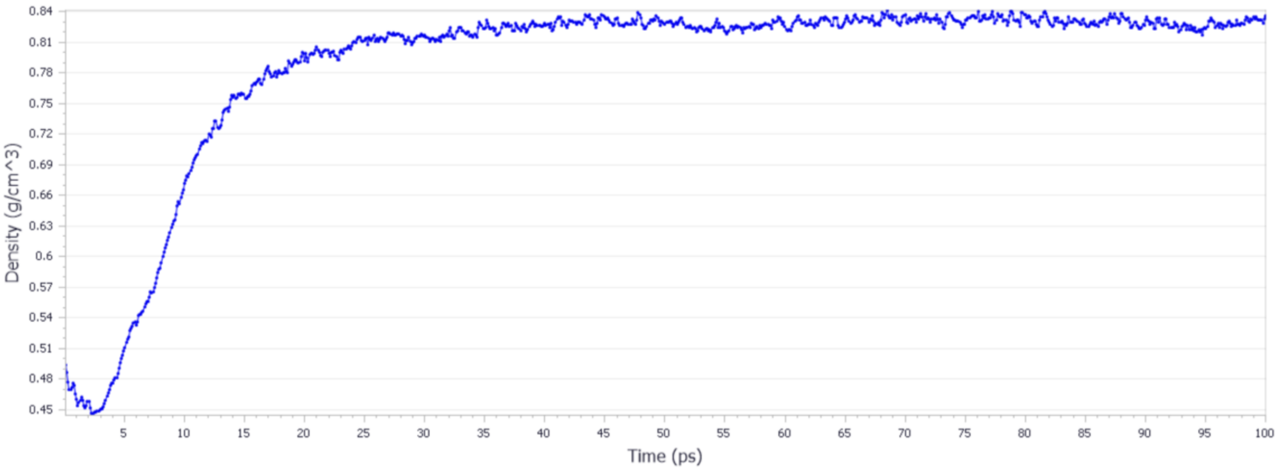

Figure S4. Evolution of the density of the polypropylene structural model during the NPT molecular dynamics run at 298 K.

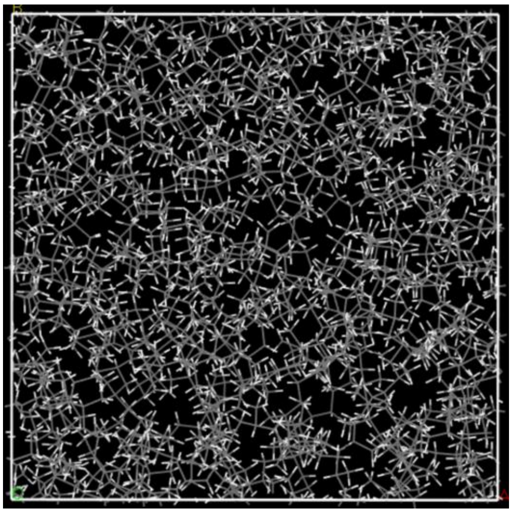

Figure S5. Polypropylene structural model after several annealing cycles 298 K to 600 K and back.

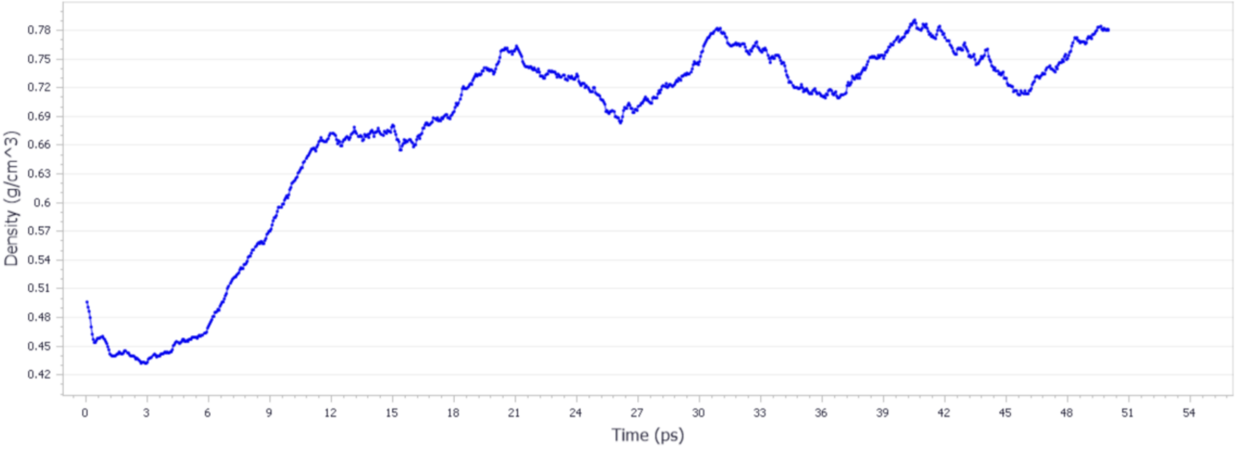

Figure S6. Evolution of the density of the PP structural model during annealing cycles.

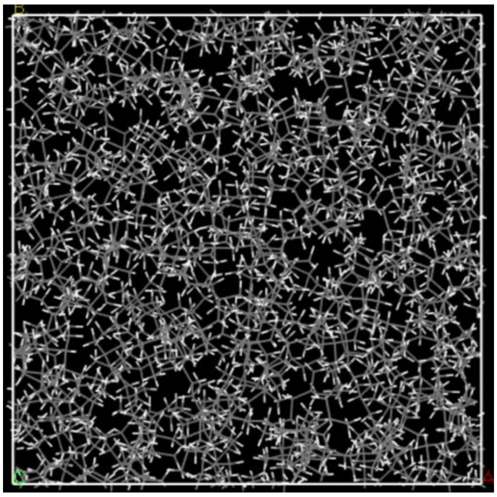

Figure S7. Polypropylene structural model after final NPT molecular dynamics run at 298 K.

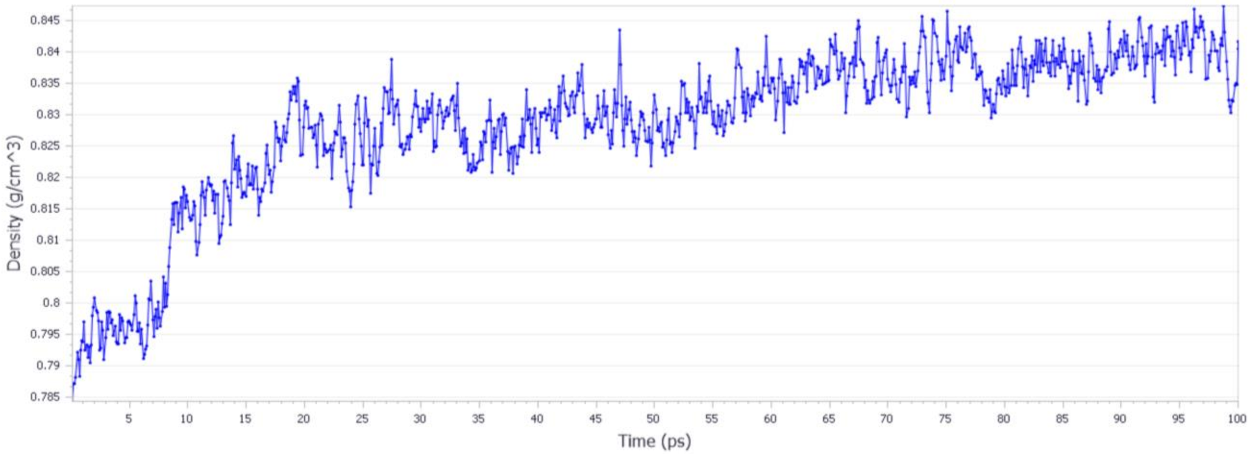

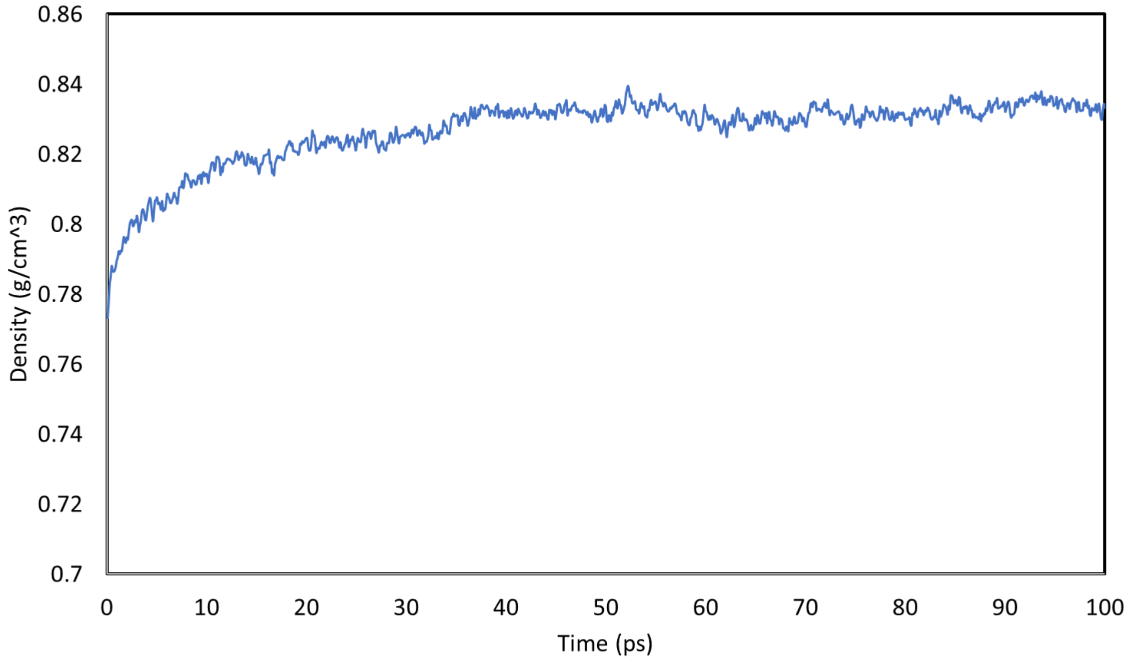

Figure S8. Top: Evolution of the density of the polypropylene structural model during the final NPT molecular dynamics run at 298 K (analogous model to figures S1 to S7). Bottom: Evolution of the density of the polypropylene structural model during the final NPT molecular dynamics run at 298 K (PP model with 50 monomers of 50 chains per unit cell).

Additional structural figures for the cellulose-PP-MAH biocomposite.

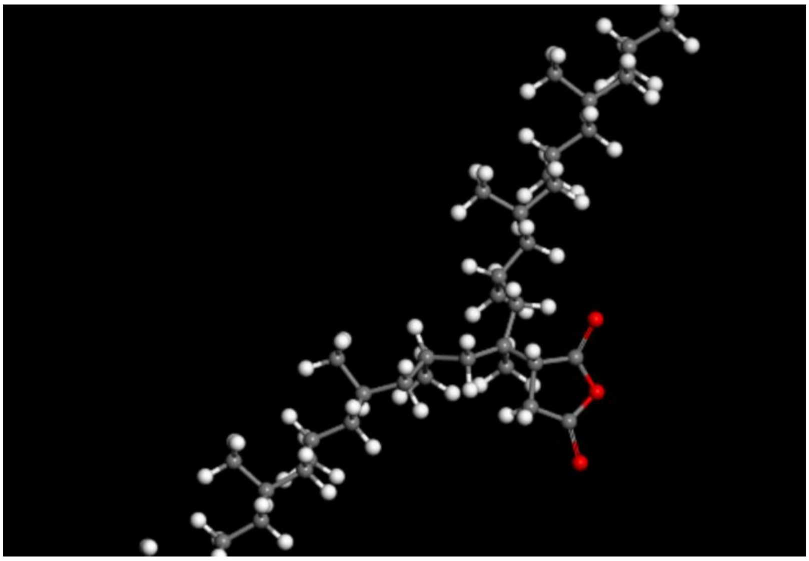

Figure S9. The position of MAH in 50 monomers long PP chain is in the middle of the chain and in the same carbon atom as the methyl group.

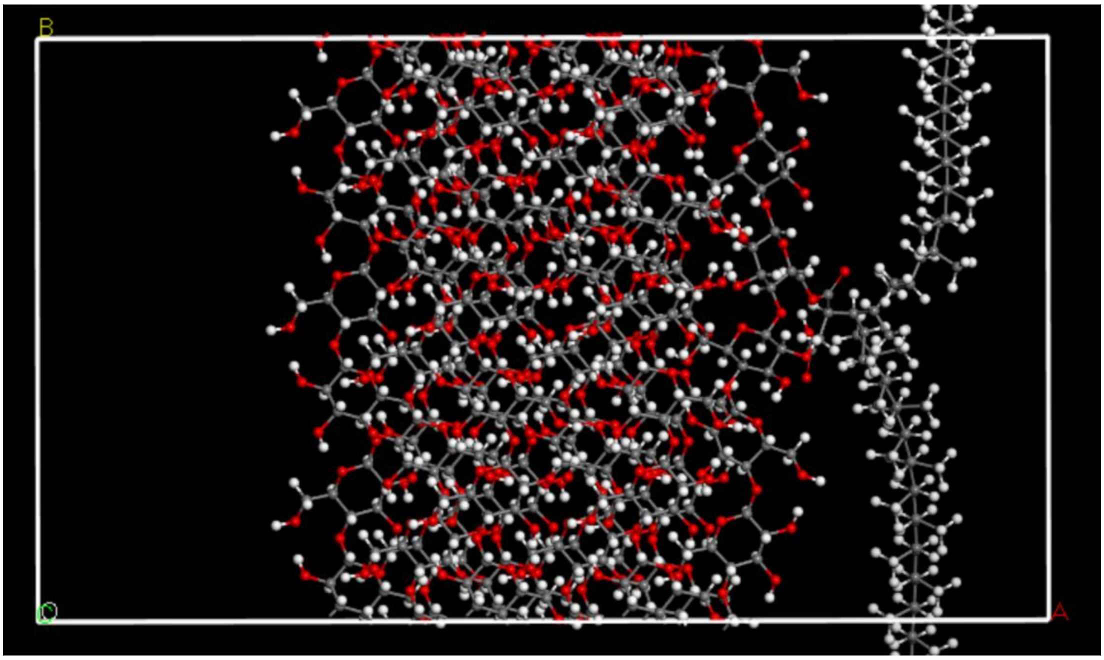

Figure S10. Side view of PP-MAH, with one MAH group in the middle of the 50 monomers long syndiotactic PP chain and in the same carbon atom as the methyl group, bonded to cellulose fibril with diameter of 2.69 nm and 10 chains of cellulose. The other PP chains included in the final cellulose-PP-MAH composite structural model are not shown in the figure.
